# Leader Humility, and Subordinates’ Organizational Citizenship Behavior and Withdrawal Behavior: Exploring the Mediating Mechanisms of Subordinates’ Psychological Capital

**DOI:** 10.3390/ijerph17072544

**Published:** 2020-04-08

**Authors:** Xiaoye Qian, Meijuan Zhang, Qiang Jiang

**Affiliations:** Business School, Sichuan University, Chengdu 610065, China; xyqian@scu.edu.cn (X.Q.); meijuanzhang@stu.scu.edu.cn (M.Z.)

**Keywords:** leader humility, psychological capital, organizational citizenship behavior, withdrawal behavior, social information processing theory

## Abstract

As a bottom-up leadership style, leader humility has received considerable attention from researchers. Among the abundant studies revealing the positive impact of leader humility on employees’ work attitude and behaviors, there is less knowledge on how leader humility influences subordinates’ organizational citizenship behavior (OCB) and withdrawal behavior. On the basis of the social information processing theory, this study proposed a cross-level mediation model and examined the direct impact of leader humility on subordinates’ OCB and withdrawal behavior. We also further explored the underlying psychological mechanism and examined the mediating effect of psychological capital on these relationships. Using a two-wave panel design and 274 employees’ questionnaire data, the empirical analysis found that: (1) leader humility was positively related to subordinates’ OCB and negatively related to subordinates’ withdrawal behavior; (2) leader humility was positively related to subordinates’ psychological capital; and (3) psychological capital played a cross-level mediating role in *the leader humility-subordinates’ OCB relationship* and *the leader humility-subordinates’ withdrawal behavior relationship*. Theoretical and practical implications, limitations, and suggestions for future research are also discussed.

## 1. Introduction

The world is becoming increasingly complex and dynamic, with rapid changes in the political, economic, market, and technological environment, and “new thinking and new approaches have become necessary for organizations to survive and to create sustainable growth and development” [1]. To achieve and maintain healthy development in such an environment, it is crucial for enterprises to have good leaders who can foster subordinates’ positive psychological strength, guide and motivate them to exhibit positive work behavior (such as organizational citizenship behavior, OCB), and reduce negative work behaviors (such as withdrawal behavior) [2]. The literature on leadership in recent years has been focusing on leader humility. According to the definition of leader humility proposed by Owens et al. [3,4], it is “(a) a manifested willingness to view oneself accurately, (b) an appreciation of others’ strengths and contributions, and (c) teachability or openness to new ideas and feedback” [3,4]. Leader humility may help foster subordinates’ positive psychological strength, as it is a bottom-up leadership style. This will ultimately influence the employees’ work behaviors. Drucker [5] pointed out that leaders should drop airs of omniscience and authority, avoid communicating in monologues, and stay humble and cautious. Owens and Hekman [4] also believe that humble leaders are better able to understand a situation and lead enterprises to create continuous and healthy development. In China, humility is highly valued by enterprises and managers, more so than in Western culture. This is because humility is considered a traditional virtue of the Chinese people [6]. Ancient proverbs such as “The humble receive benefit, while the conceited reap failure” and “Be humble” have been constantly repeated and praised by each generation, making humility an integral aspect of the Chinese culture. Jack Ma, founder of Alibaba Group, said that humility is a necessary quality for a successful manager [7]. Additionally, with the deepening of China’s reform and opening up and the rapid development of its economy, humble leaders can provide employees with opportunities to develop their strengths, promote their healthy development, and, as a result, help enterprises to better cope with the rapidly changing business environment. Therefore, leader humility is becoming increasingly important within the framework of a global economy that emphasizes sustainable growth and healthy development.

The important role of leader humility in management efficacy has drawn increasing attention in academic and business circles, and the effectiveness of leader humility in organizations has been verified. Recent studies find that the humble leader is able to set an example for employees’ positive work behavior, helping them to grow in a bottom-up manner [4] and improve individual psychological empowerment [8], job satisfaction [3,9], organizational identity [10], work engagement [11,12], creativity [13], team performance [14], and employee job performance by encouraging team members to establish harmonious interpersonal relationships [3,11]. Despite these advances, little research exists in terms of investigation of the direct impact of leader humility on subordinates’ extra-role behaviors, captured by the OCB [6]. A noticeable omission in the existing body of research is the relationship between leader humility and subordinates’ withdrawal behavior. In addition, although empirical evidence reveals a positive association between leader humility and employees’ OCB [6], the psychological mechanism underlying this relationship is still unclear.

To address these research gaps, we try to identify how leader humility affects subordinates’ OCB and withdrawal behavior. Research on social information processing theory [15] will provide a theoretical framework for this study. From the standpoint of this theory, individuals rely on social information cues to understand and judge their environment. Social context can not only directly help individuals to construct and interpret events, it can also indirectly draw individuals’ attention to certain kinds of information, and consequently shape their attitudes and psychological state. In the work environment, the humble leader serves as a key social information source whose attitude and behavior provide important social information clues to subordinates and influence their judgment of the work environment, consequently influencing their psychological state, captured by psychological capital (PsyCap) [14]. Thus, we suggest that subordinates’ PsyCap as a positive psychological state of development will play a mediating role in which leader humility relates to subordinates’ OCB and withdrawal behavior.

Rego et al.’s [14] study examined the above-mentioned mechanism. They found that team PsyCap plays a mediating role in the relationship between leader humility and team performance. Different from Rego’s work, our study focused on the social impact of the leader on individual employees’ PsyCap. We explored the cross-level mediating mechanism of subordinate’s PsyCap in the relationship between leader humility and subordinates’ OCB and withdrawal behavior. 

The remainder of this study is organized as follows: in Section 2, we review the relevant theories, develop a theoretical model, and propose four hypotheses. In Section 3 and Section 4, the method and results of the empirical analysis are examined. The theoretical contributions and managerial suggestions are discussed in Section 5. In Section 6, we summarize the main achievements and research results.

## 2. Literature Review and Hypotheses

### 2.1. Leader Humility, OCB, and Withdrawal Behavior

The word humility derives from the Latin term humus, meaning “earth”, and humi, meaning “on the ground” [4]. Humility as a concept has been called “the fertile soil from which all other virtues grow” [14,16,17,18]. Accordingly, Owens and Hekman [4] defined leader humility behavior as “leading from the ground”. Different from “top-down” leadership (i.e., authoritarian leadership, paternalistic leadership, or transformational leadership), leader humility is a “bottom-up” leadership style [13,19] that is distinctly characterized in the following three aspects: (1) a willingness to see the self accurately—humble leaders have the courage to admit their own shortcomings and mistakes in front of their subordinates, pursue a more objective appraisal of strengths and limitations, and not feel ashamed to ask for help and learn from their subordinates; (2) a genuine appreciation of subordinates’ strengths and contributions—humble leaders often publicly express recognition and praise for their subordinates for their efforts, strengths, and excellent working abilities, without feeling threatened by them; and (3) modeling teachability—humble leaders show openness to new ideas and information, prefer to listen to and think carefully about subordinates’ opinions before speaking, and are very receptive to others’ feedback on their current course of action [3,4]. Although previous studies have confirmed the positive impact of leader humility on employees [3,4,8,9,10,11,12,13], there is little empirical understanding of how leader humility influences subordinates’ development of OCB and reduction of withdrawal behavior.

Organizational citizenship behavior (OCB) refers to a series of constructive and voluntary behaviors in employees that are not explicitly stipulated by the job description and not included in the formal reward system in the organization, but can promote the effective functioning of the organization [20,21]. OCB is a typical extra-role behavior that is not included in the scope of reward and punishment standards of the organization. OCB can not only “serve as an effective means of coordinating activities between team members and across work groups”, but also “enhance the organization’s ability to attract and retain the best people by making it a more attractive place to work” and “enhance an organization’s ability to adapt to environmental changes” [22]. Previous research indicates that OCB is negatively related to employee turnover intention and plays a positive role in organizational performance [22,23]. Therefore, it is of great importance for the organization to understand how to effectively motivate employees to engage in more OCB. Prior studies have demonstrated that leaders’ ethical behavior and supportive leadership lead to more frequent OCB in employees [24,25]; yet, discussions of the effect of leader humility on OCB is rare [26]. This is the research gap our study tries to fill. 

Withdrawal behaviors were defined by Hanisch and Hulin [27] as a set of negative behaviors that employees enact to avoid work tasks under dissatisfying organizational situations. Examples of employee withdrawal behaviors include withholding efforts at work, lateness, absenteeism, social loafing, and turnover [28,29,30]. Relevant studies show that such behaviors are widespread in organizations and have strong destructive effect on an enterprise’s healthy development. For example, Sagie et al. [28] used data from a middle-sized high-tech company in Israel to calculate that the economic loss due to employee withdrawal behavior was as high as USD 2.8 million (accounting for 16.5% of the company’s pre-tax income). Other recent empirical studies also showed the negative effect of employees’ withdrawal behaviors on their job performance [31,32]. Given that employee’s withdrawal behavior has such a negative impact on the organization, the way in which to effectively curb employee’s withdrawal behavior is exactly what we try to explore in this article.

According to social information processing theory, individuals tend to judge and understand their work environments by processing social information and then construct and interpret events in the workplace. Such interpretations will, in turn, shape their work attitudes and behaviors [14]. Previous studies suggest that leaders are crucial sources of social information because of their high status and direct interactions with their subordinates [33,34]. Subordinates tend to gather useful information from their leaders’ statements and behaviors to shape the perception of the work environment [13,35]. As such, when humble leaders express appreciation and respect toward their subordinates and encourage them to give full play to their own light, it will arouse strong gratitude and trust in subordinates [3]. In turn, this increases subordinates’ OCB. Moreover, as leader humility is a bottom-up leading approach, one of its features is that the leader pays more attention to employees’ welfare and satisfying their needs [19]. Such leaders’ behaviors can enhance employees’ willingness to exhibit more OCB by arousing a strong sense of reciprocity and social exchange [6].

Similar to the above mechanism, we propose that leader humility may attenuate subordinates’ withdrawal behaviors. Previous studies have revealed that employees tend to exhibit withdrawal behaviors when they experience less organizational support, feel unimportant, are not challenged in their job or are dissatisfied with the work conditions, or experience a lack of trust [27,36]. By contrast, leader humility can attenuate employees’ withdrawal behavior by creating a safe work environment [13]. Specifically, humble leaders admit their own shortcomings and mistakes in front of their subordinates, express appreciation toward their subordinates, and encourage subordinates to try new methods to fulfill tasks [3,4], which makes employees feel that their work is valued and important. Furthermore, humble leaders respect and trust their subordinates and show openness to new ideas and information [3,4,14], which makes employees feel psychologically safe when engaging in challenging work tasks [13]. In particular, humble leaders provide support and help when employees encounter difficulties [7], which will make subordinates less likely to exhibit withdrawal behaviors “because the perception of a safe climate allows them to overcome the anxiety and fear of failure” [13]. Therefore, leader humility may curb subordinates’ withdrawal behaviors.

On the basis of the above analysis, we proposed the following hypotheses:

 **Hypothesis1.**  Leader humility is positively related to subordinates’ OCB.

 **Hypothesis2.**  Leader humility is negatively related to subordinates’ withdrawal behavior.

### 2.2. Leader Humility and Subordinates’ PsyCap

PsyCap refers to “the general core psychological element of an individual’s positive psychological state of development (p.2)” [37]. It consists of the four dimensions of self-efficacy (having the confidence to take on and put in the necessary effort to succeed at challenging tasks), optimism (making a positive attribution about succeeding now and in the future), hope (persevering toward goals and, when necessary, redirecting paths to goals in order to succeed), and resilience (when beset by problems and adversity, sustaining and bouncing back and even beyond to attain success), emphasizing the strength and positive psychological power of the person [38]. Very simply, PsyCap can be viewed as “who you are” and “what you can become in terms of positive development” [39] and is differentiated from human capital (“what you know”), social capital (“who you know”), and financial capital (“what you have”) [1]. Some studies have confirmed that individual PsyCap has positive effects on individuals’ job attitudes [40,41], work behaviors [42], and performance [1,40]. In addition, PsyCap has been shown to predict satisfaction with work, health, relationships, and life in general [43,44]. As a role model in an organization or team, the leader plays an effective role in guiding their subordinates to develop their self-confidence, hope, optimism, and resilience [45].

We propose that leader humility encourages and promotes subordinates’ PsyCap. A humble leader can “provide positive feedback on team performance; encourage new ways of accomplishing the work; create a sense of validation of strengths; and foster a positive, growth-based, developmental paradigm about organizational life” [14], which contributes to the development of subordinates’ positive PsyCap. Specifically, humble leaders show several characteristics and behaviors that contribute to developing subordinates’ PsyCap: (1) humble leaders attach importance to the value and appeal of employees and are more willing to provide subordinates with work support and help, which can motivate employees to work hard and perform at their best [3,11,13]. Subordinates therefore have more confidence to put in the necessary effort to succeed at challenging tasks (self-efficacy) [1]; (2) humble leaders believe that individuals’ abilities can be shaped, and therefore they encourage subordinates to actively embrace challenges, explore new ways to solve problems, and persevere in their goals, and, when necessary, redirect paths to goals to succeed (hope); (3) humble leaders can sincerely appreciate others’ efforts, strengths, and contributions, and thus they are keen to give praise and rewards timeously when their subordinates perform well, which can help subordinates to develop a positive attribution (optimism) for succeeding now and in the future; (4) humble leaders not only tolerate their subordinates’ failures and mistakes, but also consider mistakes as a normal and even a beneficial part of learning [4], and therefore, when subordinates are beset by problems and adversity, the humble leader tends to share responsibility and encourage them to continue to try. Accordingly, subordinates can sustain their effort, bounce back, and reach success (resilience). In summary, leader humility has a significant impact on the four components of subordinates’ PsyCap: self-efficacy, optimism, hope, and resilience. Rego et al.’s [14] research has found that leader humility has a positive influence on team’s PsyCap. Therefore, we propose the following hypothesis:

 **Hypothesis3.**  Leader humility is positively related to subordinates’ PsyCap.

### 2.3. The Mediating Role of Subordinates’ PsyCap

We also propose that subordinates’ positive PsyCap has an important driving effect on their OCB and a significant attenuating effect on withdrawal behavior. This means that subordinates’ PsyCap may play a mediating role in the relationship between leader humility and subordinates’ OCB/withdrawal behavior. First, several previous studies support the promoting effect of positive PsyCap on employees’ extra-role behaviors [10,41,42,46,47,48]. For example, Qian et al. [46] found that employees with higher self-efficacy are less reluctant to speak up; Norman et al.’s [10] research demonstrated that positive PsyCap can promote employees to engage in more OCB and fewer deviance behaviors; and Avey et al.’s [48] meta-analysis supported the idea that PsyCap is positively related to desirable OCB behaviors, and negatively related to undesirable behaviors (turnover and deviance). Consistent with these arguments, we propose the idea that subordinates’ perceptions of leader humility may create positive work conditions necessary for subordinates’ PsyCap to flourish, which, in turn, will promote subordinates to engage in more OCB [10,48] and fewer withdrawal behaviors [48].

In summary, by providing support and help to their subordinates, trusting and appreciating their abilities and efforts, and tolerating their failures and mistakes, leader humility will be conducive to promoting and developing subordinates’ PsyCap, thereby causing subordinates to show more OCB and reduce withdrawal behavior. We therefore propose the following hypotheses:

 **Hypothesis4a.**   PsyCap mediates the relationship between leader humility and subordinates’ OCB.

 **Hypothesis4b.**  PsyCap mediates the relationship between leader humility and subordinates’ withdrawal behavior.

**Figure 1:**
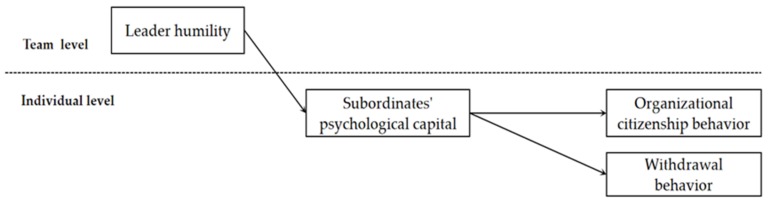
Theoretical model.

Figure 1 demonstrates our theoretical model, which includes hypotheses 1–4.

## 3. Materials and Methods

### 3.1. Group Reached and Procedure

The data used in this study were collected from four large state-owned medical institutions located in southeast China. We had initially received a list of 13 medical institutions from the municipal government. Using convenience sampling, we randomly contacted half of the organizations (six medical institutions) on that list, and finally reached an agreement with four of them. A total of 355 employees from 66 teams participated in the two-wave survey. The data collection phase lasted 2 months and was aimed at reducing potential estimation bias arising from common method biases. The data collection procedure was as follows: first, two weeks before the first survey—with the help of human resource (HR) managers—we obtained the participants’ demographic information, including age, gender, educational level, and work experience. During the first phase (time 1), we distributed questionnaires to participants in envelopes and asked them to return the completed questionnaires directly to our research assistant. In this wave, employees rated their manager’s leader humility and their own PsyCap, and shared their demographic information. We cross-checked the participants’ self-reported demographic information with information from their HR departments. During the second phase (time 2), the participants answered questions on OCB and withdrawal behavior. After excluding the invalid questionnaires such as those lacking demographic information and data and those from the two waves that could not be matched, the final sample consists of 274 effective observations (a return rate of 77.18%). Among the participants, 73% were female and 55% had at least a bachelor degree or higher. In terms of the average age and job tenure, the respondents were 37 years old and had been working at their organization for 11 years.

### 3.2. Measures

We used a five-point Likert scale (from 1 = “strongly disagree” to 5 = “strongly agree”) for all scales. Because the measurements were originally developed in English, we followed strict translation and back-translation procedures to ensure that the original scales can be used in a Chinese context.

#### 3.2.1. Leader Humility

Leader humility was measured with an 11-item scale developed and validated by Owens et al. [11]. Example items are “My leader admits it when he or she doesn’t know how to do something”, “My leader acknowledges when others have more knowledge and skills than themselves”, “My leader shows a willingness to learn from others”, and “My leader admits it when he or she makes mistakes” (Cronbach’s α = 0.96).

#### 3.2.2. PsyCap

PsyCap was measured with a 24-item scale developed by Luthans et al. [49]. Data were collected via participant self-reporting. The PsyCap scale consists of four dimensions: self-efficacy, hope, resilience, and optimism. Example items are (a) self-efficacy: “I feel confident in helping set targets/goals in my work area”; (b) hope: “I can come up with different ways to achieve my current goal”; (c) resilience: “When I have a setback at work, I can recover from it and move on”; and (d) optimism: “I always look on the bright side of things regarding my job”. The Cronbach’s alpha for PsyCap was 0.97. As for each dimension of the PsyCap, the Cronbach’s alpha was 0.91 for self-efficacy, 0.93 for hope, 0.93 for resilience, and 0.92 for optimism.

#### 3.2.3. OCB

OCB was measured using Williams and Anderson’s [29] eight-item scale. Example items are “I do what my job does not require me to do, but is conducive to improving the image of my organization”, “I take the initiative to learn the development of my organization”, and “When others criticize my organization, I refute them” (Cronbach’s α = 0.92).

#### 3.2.4. Withdrawal Behavior

Withdrawal behavior was measured with an eight-item scale developed by Roznwski and Hanisch [50]. Example items are “Using the work phone for personal calls”, “Making excuses to get of regular working meetings”, and “Letting others do your work for you” (Cronbach’s α = 0.88).

#### 3.2.5. Control Variables

We controlled for gender, age, educational level, and job tenure at an individual level and for team type and team size (i.e., number of teams) at the team level because of their potential impact on employees’ attitudes and behaviors [11,13,20]. Gender was coded as “0” for male and “1” for female; age and job tenure were measured in years; and educational level was a categorical variable, going from 1 to 4 representing, respectively, “high school and below”, “technical secondary school”, “junior college”, and “college and above”. We also used the team type function as a team-level control variable going from 1 to 4, respectively indicating “outsourcing business unit”, “logistics department”, “administrative department”, and “business unit”.

### 3.3. Analytic Strategy

As our data were nested in teams (i.e., multiple subordinates in the same team share the same leader), a multi-level confirmatory factor analysis (CFA) [51] was used to examine the properties of our measures and hierarchical linear modeling (HLM) [52] was used to test hypotheses with Stata14 software. When testing the multilevel mediation hypothesis, we used a maximum likelihood estimation and robust standard errors.

### 3.4. Aggregation of Team-level Variable

It is necessary to check the viability of the team-level variable, which is leader humility. We calculated the mean r_wg_ value for leader humility to be 0.97, which is higher than the conventionally acceptable r_wg_ value of 0.70 [53,54], showing a satisfactory internal consistency. Additional evidence was obtained following the suggestions of Bliese [55], the interrater reliability index (ICC1), and the reliability of group mean index (ICC2). For leader humility, the ICC1 was 0.03 and the ICC2 was 0.90. Taken together, these results support the aggregation of leader humility.

## 4. Results

### 4.1. Confirmatory Factor Analysis and the Test of Common Method Variance

Before testing the proposed hypotheses, we conducted a CFA to examine the discriminant validity of the four latent variables: leader humility, PsyCap, OCB, and withdrawal behavior. The relevant statistical indicators of model goodness of fit are shown in Table 1, the hypothesized four-factor model fitted the data better (χ2 = 3423.988, RMSEA = 0.081, CFI = 0.818, TLI = 0.810). The factor loadings of all items were above 0.55. Taken together, these statistics supported the variables’ discriminant validity.

Additionally, we conducted a CFA analysis for each of the instruments to examine their reliability and validity. Generally, if average variance extracted (AVE) is greater than 0.5, and composite reliability (CR) is greater than 0.7, it means the instruments have good convergent validity and composite reliability [56,57]. In this study, the values of AVE for leader humility, PsyCap, OCB, and withdrawal behavior were all greater than 0.5, and the CR values and Cronbach’s alpha were all higher than 0.7, which meant that all the scales used in this study had good reliability and convergent validity.

Because the questionnaires were self-reported by employees, we tested potential common method variance by using Harman’s single factor test [58]. As shown in Table 1, the single factor model had a poor fit with the data (χ2 = 7459.023, RMSEA = 0.136, CFI = 0.486, TLI = 0.465). Furthermore, we performed an exploratory factor analysis (EFA) analysis of all four variables and found the first factor only explained 30.58% of the variance. Therefore, it can be concluded that common method bias was not an issue in this study.

### 4.2. Descriptive Statistics and Correlations

Table 2 presents a summary of the descriptive statistics and correlations among all the individual-level and team-level variables. As shown in Table 2, subordinates’ PsyCap was positively correlated with subordinates’ OCB (*r* = 0.66, *p* < 0.01) and negatively correlated with subordinates’ withdrawal behavior (*r* = −0.16, *p* < 0.01).

### 4.3. Hypothesis Testing

We use a hierarchical regression analysis to test hypotheses 1–4 and the results are shown in Table 3. To test the cross-level effect among variables, we followed the suggestion of Liao and Zhuang [59], and both individual-level and team-level variables were mean-centered before testing [60,61].

First, we examined the null model, which only included the control variables. The results are reported in Table 3, columns 2 and 5.

Hypothesis 1 predicted that leader humility would be positively related to subordinates’ OCB. As displayed in model 1 (Table 3), the relationship between leader humility and subordinates’ OCB was significantly positive (*β* = 0.51, *p* < 0.01). Therefore, hypothesis 1 was supported.

Hypothesis 2 predicted a negative relationship between leader humility and subordinates’ withdrawal behavior. As showed in model 4 (Table 3), leader humility was negatively related to subordinates’ withdrawal behavior (*β* = −0.27, *p* < 0.05). Therefore, hypothesis 2 was supported.

Hypothesis 3 predicted a positive relationship between leader humility and subordinates’ PsyCap. As shown in model 6 (Table 3), the relationship between leader humility and subordinates’ PsyCap was significantly positive (*β* = 0.47, *p* < 0.01). Therefore, hypothesis 3 was supported.

Hypotheses 4a and 4b predicted the cross-level mediating role of subordinates’ PsyCap on the relationship between leader humility and subordinates’ OCB/withdrawal behavior. We used HLM [52] in conjunction with the mediation testing procedure proposed by Baron and Kenny [62] to test this hypothesis. First, in testing hypotheses 1 and 2, the results showed that leader humility (independent variables as X) was significantly related to OCB and withdrawal behavior (dependent variable as Y). Next, in testing hypothesis 3, the results showed that the independent variable leader humility had a significant positive relationship with the mediator, subordinates’ PsyCap (as M). In short, the first two conditions of the mediation test (X→Y, X→M) were satisfied [62]. The final step for testing the mediating effect was to regress OCB/withdrawal behavior simultaneously on leader humility and subordinates’ PsyCap. The results in model 2 and model 5 (Table 3) showed that subordinates’ PsyCap was positively related to subordinates’ OCB (*β* = 0.66, *p* < 0.01) but negatively related to subordinates’ withdrawal behavior (*β* = −0.12, *p* < 0.1). At the same time, the effect of leader humility on subordinates’ OCB become weaker (*β* = 0.20, *p* < 0.05) when the subordinates’ PsyCap was included; the effect of leader humility on subordinates’ withdrawal behavior became insignificant (*β* = −0.21, ns) when the subordinates’ PsyCap was included. Therefore, subordinates’ PsyCap not only partially mediated the relationship between leader humility and subordinates’ OCB, but also completely mediated the relationship between leader humility and subordinates’ withdrawal behavior. Thus, hypotheses 4a and 4b were both supported.

## 5. Discussion

### 5.1. Theoretical Implications

This study focused on the underlying influence of leader humility on subordinates’ OCB and withdrawal behavior. Applying a two-phrase research design with a sample of 274 employees in 58 teams from four stated-owned medical institutions located in southeast China, our four hypotheses were all supported. In particular, we found that: (1) leader humility had a positive impact on subordinates’ OCB; (2) leader humility had a significant attenuating effect on subordinates’ withdrawal behavior; and (3) subordinates’ PsyCap played a cross mediating role in the relationships between *leader humility–subordinates’ OCB* and *leader humility–subordinates’ withdrawal behavior*, respectively.

The findings of this study expand the knowledge in the study area of leader humility and positive PsyCap in the following aspects:

First, this study represented the first attempt to integrate and test the influence of leader humility on subordinates’ positive work behaviors (OCB) and negative work behaviors (withdrawal behavior), which constitutes a notable contribution to literature on leader humility. By examining the impact of leader humility on subordinates’ positive and negative work behaviors, we proved that leader humility has a driving effect on subordinates’ OCB, meanwhile having an attenuating effect on subordinates’ withdrawal behavior. Studies on the impact of leader humility have produced some significant results, and point to increased psychological empowerment, job satisfaction, organizational identity, job involvement, job performance, and creativity [3,7,8,9,10,13]. In particular, from the perspective of interpersonal relationships, Mao et al. [6] indicated that leader humility could promote subordinates’ voice and helping behaviors by allowing the leader to build a close relationship with them. However, there are few studies that have focused on the impact of leader humility on subordinates’ extra-role behavior (OCB) within the Chinese context. Furthermore, to the best of our knowledge, there are no studies that have explored the influence of leader humility on subordinates’ withdrawal behaviors. Our research filled this gap and explored the driving effect of leader humility on subordinates’ OCB and its attenuating effect on withdrawal behavior, thereby enriching the research on the effectiveness of leader humility.

Second, this research found a new antecedent that affects employee PsyCap, which expands our understanding of the cultivating role that leadership plays in developing individuals’ PsyCap. Moreover, our research also contributes to occupational health psychology. Previous studies have shown that PsyCap facilitates positive cognitive appraisals of work and life, and plays a positive role in well-being, and in satisfaction with work, health, and life [43,44]. Hence, PsyCap, as an individual’s positive and healthy psychological state of development, is of great significance for employees [40,41,42]. Scholars have examined several different leadership types and how they influence subordinates’ PsyCap. For example, Wang et al. [13] found that transformational leadership could enhance employees’ PsyCap by influencing their cognitive processes, whereas Bouckenooghe’s research [45] demonstrated that ethical leadership has a positive effect on employees’ PsyCap via a role model effect. How leader humility—as a new “bottom-up” leadership style—affects subordinates’ psychological capital has not been fully discussed. Building on recent research that has documented the influence of leader humility on collective character strengths in the teams they lead [14], our research revealed that leader humility can effectively foster subordinates’ positive PsyCap, which provides a theoretical reference for further understanding the influential effect of leader humility. At the same time, the discovery of a positive relationship between leader humility and subordinate’s PsyCap enriches the literature on the occupational health psychology.

Finally, our research explored the underlying psychological mechanism of the relationships between leader humility and subordinates’ OCB and withdrawal behavior, on the basis of social informational processing theory. By reiterating the findings of Jeung and Yoon [8], who explored the psychological mechanisms between leader humility and employee attitudes and behavior, we proposed and tested an important psychological mechanism, this being the fact that the driving effect of leader humility on subordinates’ OCB and its attenuating effect on withdrawal behavior are both channeled by subordinates’ PsyCap. Specifically, leader humility can promote subordinates’ self-efficacy, hope, optimism, and resilience, which are four factors of their PsyCap, and then lead to higher OCB and less withdrawal behavior. The demonstration of the cross-level mediating effect of subordinates’ PsyCap enriches the literature on the potential influence of humble leadership on subordinates’ healthy work behaviors and makes a new contribution to the positive psychology literature.

### 5.2. Practical Implications

These findings may have several practical implications for managers and enterprises.

On one hand, our research showed that leader humility plays an important role in promoting subordinates’ positive work behavior (OCB), and that it has a significant attenuating effect on subordinates’ negative work behavior (withdrawal behavior). As a traditional virtue of the Chinese people, leader humility has been confirmed by our research as an important leadership trait that can be learned and developed [14]. Thus, it is of great significance to organizations, especially Chinese enterprises. Therefore, enterprises should focus on selecting and cultivating humble leaders. Moreover, leadership training and development programs should be provided to help leaders understand the importance of humility, to develop their humility, as well as to encourage them to practice humble behavior in their organizations.

On the other hand, this study revealed that leader humility can improve subordinates’ PsyCap, which, in turn, promotes subordinates’ extra-role behaviors. Therefore, leaders should strive to incorporate more humble behaviors in their daily work to promote subordinates’ PsyCap, such as lowering their postures; admitting their shortcomings and mistakes; paying attention to subordinates’ strengths; praising them for good work; giving support and assistance when subordinates are facing challenges; and showing trust, respect, and willingness to acquire new knowledge and skills. As a result, the subordinates feel supported, recognized, appreciated, rewarded, and treated fairly, and PsyCap is likely to thrive and yield desired outcomes [43]. In addition, organizations could adopt managerial interventions to improve employees’ PsyCap. For example, organizational managers should strive to create a positive and psychologically safe culture and implement relevant training for developing the PsyCap of employees [43]. These can foster and develop subordinates’ positive PsyCap, and, as a result, motivate employees to increase their OCB and reduce withdrawal behavior.

### 5.3. Limitations and Future Directions

Our study has several limitations that should be addressed in future research.

First, to avoid common method bias, we collected longitudinal data at two times, which could weaken the influence of common method bias. However, as the independent variable (leader humility) and the mediating variable (PsyCap) in our model were both self-evaluated by employees in the first stage, although the result showed that common method bias was not a major problem in this study, the theoretical model may still have been influenced by common method bias. Using objective data to measure team and individual variables or collecting multi-wave panel data will undoubtedly further enhance the persuasiveness and application value of our study. In the future, it would be better to collect multi-wave panel data from employee self-assessment and leaders or peer evaluation.

Second, although our research addresses *the leader humility–subordinates’ PsyCap–subordinates’ OCB/withdrawal behavior* relationship, we did not consider how contextual factors may change the relationship. In future studies, moderating factors such as team climates and group structure should be considered for a deeper understanding of the boundary conditions of the leader humility–subordinates’ PsyCap-subordinates’ OCB/withdrawal behavior relationship.

Third, although the sample of 58 teams and 274 employees in this study was obtained from four different medical organizations, these organizations still belong to the same industry. This research design helped us to eliminate interference due to industry differences and to improve the internal validity of the research by sacrificing the external validity of the research to some extent. However, the research design may cause difficulty in externalizing our research conclusion. Therefore, future studies should further test the proposed theoretical model in the context of other industries and different types of enterprises.

Last, the effect of variables such as years of professional experience (both in leadership positions and as a member of the work team) and gender (also in relation to both professional roles: leader/team member) were not sufficiently explored in this research. Future studies would benefit from considering these variables. Furthermore, qualitative studies are necessary to deepen research on leader humility and its results at the team level.

## 6. Conclusions

On the basis of social information processing theory, this study proposed a cross-level mediation model, and tested the importance of channeling the effect of subordinates’ PsyCap in the relationships between leader humility and subordinates’ OCB and withdrawal behavior. Our empirical analysis showed that leader humility has a driving effect on subordinates’ OCB, while having an attenuating effect on subordinates’ withdrawal behavior. In addition, our research also revealed that leader humility can effectively foster subordinates’ positive PsyCap. Moreover, subordinates’ PsyCap plays a mediating role in these relationships. We hope that this study will encourage future researchers to examine the PsyCap-based mechanisms by considering boundary conditions in which leader humility influences subordinates’ work outcomes.

## Figures and Tables

**Table 1:** Results of confirmatory factor analysis.

Model	χ^2^	df	χ^2^/df	TLI	RMSEA	CFI
1-factor model ^a^	7459.023	1224	6.094	0.465	0.136	0.486
2-factor model ^b^	5550.807	1223	4.537	0.628	0.114	0.643
3-factor model ^c^	4699.670	1221	3.849	0.701	0.102	0.713
4-factor model ^d^	3423.988	1218	2.811	0.810	0.081	0.818

Notes: ^a^ leader humility + psychological capital (PsyCap) + organizational citizenship behavior (OCB) + withdrawal behavior; ^b^ leader humility + OCB + withdrawal behavior, PsyCap; ^c^ leader humility, OCB + withdrawal behavior, PsyCap; ^d^ leader humility, OCB, withdrawal behavior, PsyCap.

**Table 2:** Mean, standard deviation, and correlation coefficient.

Variable	*M*	*SD*	1	2	3	4	5	6	7
Individual-level									
1. Gender	0.27	0.44	—						
2. Age	37.05	8.83	0.20 ^***^	—					
3. Educational level	1.66	0.87	0.10	0.33 ^***^	—				
4. Job tenure	11.35	8.71	0.03	0.68 ^***^	0.22 ^***^	—			
5. PsyCap	3.65	0.61	0.03	0.02	−0.04	−0.00	(0.97)		
6. OCB	3.59	0.65	0.04	−0.01	−0.01	−0.07	0.66 ^***^	(0.92)	
7. Withdrawal behavior	1.76	0.69	0.13 ^**^	−0.04	−0.05	−0.06	−0.16 ^***^	−0.15 ^**^	(0.88)
Team-level									
1. Team type	2.06	0.83	—						
2. Team size	8.12	3.52	0.26 ^***^	—					
3. Leader humility	3.38	0.41	−0.13 ^**^	−0.43 ^***^	(0.96)				

Notes: *N*_individual_ = 274, *N*_team_ = 58. *** *p <* 0.01; ** *p <* 0.05. OCB = organizational citizenship behavior, M refers to mean, SD refers to standard deviation. Cronbach’s alphas appear in parentheses within the diagonal.

**Table 3:** Hierarchical linear modeling (HLM) results of the hypothesized relationships.

Variable	OCB			Withdrawal Behavior			PsyCap
	Null Model	Model 1	Model 2	Null Model	Model 4	Model 5	Model 6
Intercept	0.02	−0.02	0.13	−0.02	−0.12	−0.15	−0.24
Individual-level							
Gender		0.00	0.00		0.27 ^***^	0.26 ^***^	−0.00
Age		0.00	0.00		−0.00	–0.00	0.00
Education level		0.00	−0.01		0.03	0.03	0.02
Job tenure		−0.01	−0.01		−0.01	–0.01	−0.00
PsyCap			0.66 ^***^			–0.12 ^*^	
Team-level							
Type of team function		−0.01	0.01		−0.06	–0.06	−0.03
Team size		−0.01	−0.02		0.02	0.02	0.01
Leader humility		0.51 ^***^	0.20 ^**^		−0.27 ^**^	–0.21	0.47 ^***^
Variance within group (σ^2^)	0.40	0.37	0.22	0.40	0.38	0.38	0.34
Variance between group (τ)	0.02	0.00	0.00	0.07	0.06	0.06	0.00
Log likelihood	−269.43	−251.00	−181.68	−280.84	−271.82	−270.21	−240.69

Notes: *N*_individua__l_ = 274, *N*_team_ = 58. *** *p <* 0.01; ** *p <* 0.05; * *p <* 0.1. PsyCap = psychological capital. All the models in the table are cross-level model.
